# The Impact of Mixed Emotions on Creativity in Negotiation: An Interpersonal Perspective

**DOI:** 10.3389/fpsyg.2018.02660

**Published:** 2019-01-11

**Authors:** Franki Y. H. Kung, Melody M. Chao

**Affiliations:** ^1^Department of Psychological Sciences, Purdue University, West Lafayette, IN, United States; ^2^Department of Management, Hong Kong University of Science and Technology, Kowloon, Hong Kong

**Keywords:** interpersonal, mixed emotions, creativity, creative solution, negotiation

## Abstract

Creativity is critical to organizational success. Understanding the antecedents of creativity is important. Although there is a growing body of research on how (mixed) emotions affect creativity, most of the work has focused on intrapersonal processes. We do not know whether contrasting emotions between interacting partners (i.e., interpersonal mixed emotions) have creative consequences. Building on information processing theories of emotion, our research proposes a theoretical account for why interpersonal mixed emotions matter. It hypothesized that mixed- (vs. same-) emotion interactions would predict higher collective creative performance. We tested the hypothesis in two-party integrative negotiations (105 dyads). We manipulated negotiators’ emotional expressions (angry-angry, happy-happy, angry-happy dyads) and measured the extent to which they generated creative solutions that tapped into hidden integrative potential in the negotiation for a better joint gain. The results overall supported the hypothesis: (i) there was some evidence that mixed-emotion dyads (i.e., angry-happy) performed better than same-emotion dyads; (ii) mixed-emotion dyads, on average, achieved a high level of joint gain that exceeded the (non-creative) zero-sum threshold, whereas same-emotion dyads did not. The findings add theoretical and actionable insights into our understanding of creativity, emotion, and organization behavior.

## Introduction

Creativity is a critical asset to organization effectiveness (Anderson et al., 2014). It catalyzes the formulation of new ideas and promotes optimal conflict resolution practices, among other positive outcomes (Kurtzberg, 1998; Liu et al., 2017). Understanding the antecedents of employee creativity is vital for organizations. The literature has documented diverse personal and situational predictors of creativity, such as personal motivation (e.g., Gong et al., 2017), creative self-efficacy (e.g., Gong et al., 2009), empowering leadership (e.g., Zhang and Bartol, 2010), and organizational climate (e.g., Ekvall, 1996). Among all, the effects of emotions on creativity have drawn increasing interest (Fredrickson, 2006; Conner and Silvia, 2015).

Recent research has just begun to explore the impact of mixed emotions. Mixed emotions refer to the simultaneous experience of positive and negative emotions (also known as “emotional ambivalence”; Larsen et al., 2001; Larsen and Stastny, 2011). The experience of mixed emotions can help employees think creatively, connecting seemingly unrelated ideas, which in turn increase creative performance (e.g., Fong, 2006). However, our understanding of the link between mixed emotions and creative outcomes is still limited. This is partly because the literature has primarily focused on the *intra*personal experience of mixed emotions (Rothman, 2017). The impact of mixed emotions on *inter*personal interactions is not well understood.

Social interactions are inevitable and are often inseparable from creative work in organizations (e.g., negotiating in a work project or collaborating as a team; Kurtzberg and Amabile, 2001). However, extant work does not directly speak to whether *inter*personal experiences of mixed emotions (i.e., a pair or a group of people who express contrasting, positive and negative, emotions) matter. Would interpersonal experiences of mixed emotions help individuals realize potential interconnectedness between unrelated ideas, bridging seemingly conflicting interests to facilitate collective creative performance?

The current research addresses this question. The answer could unveil a new perspective in understanding creativity. Building on information processing theories of emotion, our research offers a theoretical account for why interpersonal mixed emotions matter. More importantly, it provides an empirical test for the effect of mixed emotions on collective creative performance—generating creative solutions in a dyadic business negotiation simulation.

### Mixed Emotions and Creativity

Emotion has information value to the self. According to feeling-as-information theory, people attend to their own felt and expressed emotions as a source of information. Positive and negative emotions are two common dimensions of emotionality that offer different information to help people think and adapt to their specific environment (Schwarz and Clore, 1996; Forgas, 2000). For instance, positive emotions, such as happiness, signal the presence or anticipation of rewards and the state of a satisfactory situation. In contrast, negative emotions, such as anger and sadness, signal the presence or anticipation of losses and the state of an unsatisfactory situation (Higgins et al., 1987; Keltner et al., 1993). Positive and negative emotions can differ as affective traits (i.e., emotionalities) and as situational states, influencing people’s judgment and cognition (Barrett et al., 2007), such as their ability to think creatively (Baas et al., 2008; Jovanovic et al., 2016).

In general, to think creatively means that one needs to be aware of and be able to combine discrepant information to form a novel and useful idea (Eysenck, 2003; Cropley, 2006; Diedrich et al., 2015). The outcome of creative thinking is a creative performance or creative solution. A novel and useful creative solution is not easy to come by. It requires people to think “outside of the box” without being bounded by usual assumptions and to form meaningful connections between seemingly unrelated ideas (e.g., Mednick, 1962). Emotions play a role in facilitating this creative thinking process. They can serve as important sources of information that shape how people interpret the relationship among information in a given situation. As mentioned, positive emotions signal anticipation of rewards and state of satisfaction, whereas negative emotions signal anticipation of losses and state of dissatisfaction (Schwarz and Clore, 1996; Schwarz, 2012). The simultaneous experience of both positive and negative emotions signals that the environment is satisfactory and unsatisfactory at the same time. These mixed emotions provide conflicting information, which can potentially be a thought-provoking experience (Fong, 2006) and offer a critical pathway through which creativity might occur.

Although people can experience mixed emotions (e.g., bittersweet, angry yet happy; Larsen et al., 2001; Tamir and Ford, 2012), it is not as common as experiencing a single focal emotion. Unlike a single emotion, experiencing mixed emotions often elicits a sense of conflict and complication. People often describe it as “feeling torn” or “conflicted” (Hong and Lee, 2010). Given that the experience of mixed- (vs. single-) emotion is relatively unusual, it motivates people to look beyond the mundane assumptions of a given situation and pre-existing non-creative options, in search for more novel interpretations and solutions (i.e., “out of the box” creative solutions; Fong, 2006). Indeed, research has shown that when operating in familiar situations, people tend to process information mindlessly (relying on readily available information and common knowledge) for quick and easy answers. However, exposure to conflicting information can trigger a more deliberative process, which reduces reliance on pre-existing schemas (e.g., Louis and Sutton, 1991) and invokes a sense of “cognitive disequilibrium” (Tadmor and Tetlock, 2006). This experience leads individuals to question their preconceptions, explore uncommon ideas, and pay attention to associations between seemingly unrelated ideas (Webster and Kruglanski, 1994; Kruglanski et al., 2009; Tadmor et al., 2012). Thus, exposure to conflicting information is an incubator for creative thinking (Fong, 2006; Rothman, 2017).

Empirical studies have provided some initial support for this argument. In a series of studies, Rees et al. (2013) showed that when one feels happy and sad simultaneously (vs. happy and sad separately), the person has increased receptivity to new perspectives. In another study, Fong (2006) randomly assigned participants to a recall a personal memory that was happy, sad, neutral, or had mixed emotions. Individuals recalling mixed emotions (vs. single emotion) later showed a greater ability to connect seemingly unrelated ideas (i.e., Remote Association Task). These findings suggest that mixed-emotions experience can enhance creative thinking.

### Mixed Emotions as an Interpersonal Phenomenon

Despite the valuable insight that mixed emotions can promote creativity, past research has uniformly conceptualized mixed emotions as an intrapersonal phenomenon that shapes creative thinking of an individual (e.g., Fong, 2006; Rees et al., 2013). However, many work contexts are inherently social, and emotional exchange is central to social interactions. The Emotion-as-social-information (EASI) model (Van Kleef, 2009) extends the feeling-as-information theories and argues that just as one’s own emotions can offer information to the self (i.e., feeling-as-information), an interacting partner’s emotional expressions can also provide information to the self. Without considering the impact of mixed emotions on the interpersonal level, our understanding of emotions and creativity remains incomplete.

During interpersonal interactions, in addition to processing emotions of the self, people process emotional expressions of their partner to inform their own thinking and behavior (Van Kleef, 2009). Positive emotional expressions can signal satisfaction and prosociality. For instance, women who displayed more positive emotions in pictures appeared as friendlier and smarter (Harker and Keltner, 2001). Customers perceived service workers who expressed happiness to provide a higher quality of service (Barger and Grandey, 2006). In contrast, negative emotional expressions can signal dissatisfaction and dislike. In a study of work teams, subordinates viewed an angry (vs. happy) leader as more dissatisfied with work progress and put more effort into their work (Sy et al., 2005). In a negotiation study, when the opponents expressed happiness, the negotiators inferred that the opponents could be easily satisfied, and therefore, conceded less to the opponents (Van Kleef et al., 2004b). To unpack the role of emotions in interpersonal contexts, we need to understand the emotional expressions of interacting partners. Thus far, most studies have focused on either the actor’s or the partner’s emotional expression. As little research has examined the emotions of both parties simultaneously, there is still much to learn about how emotions interplay in interpersonal situations.

The two emotion theories discussed above—feeling-as-information theory and the EASI model—suggest that there are (at least) two sources of emotions operating simultaneously in an interpersonal situation: emotions expressed by the self and emotions expressed by the partner. Both self- and partner-expressed emotions have informational value. They can converge (e.g., both partners show happiness or anger; same positive or negative emotions) or they can contrast (e.g., one partner shows happiness and the other shows anger; different positive and negative emotions).^1^ Both self- and partner-expressed emotions are essential information sources, affecting the thinking and behavior of the parties involved. This insight speaks to the need for a framework to understand and to test mixed emotions in interpersonal contexts. The current work serves these aims. In the following, we theorize the impact of contrasting emotions expressed between two interacting partners on creative performance.

#### An Interpersonal Mixed-Emotions Theoretical Framework

There are many examples of social situations in which emotions expressed by the parties involved can contrast with one another (Niven, 2016). In romantic relationships, couples can express different emotions on the same issue. Recognizing and connecting emotions expressed in a couple’s interaction can be a form of couple therapy (Johnson, 2004). In the workplace, negotiators express varying emotions in a negotiation, which can affect how they negotiate a deal (e.g., happier negotiators tend to build more long-term business relationships; Van Kleef et al., 2004b; Kopelman et al., 2006). Members of a team can differ in their feelings toward a task and toward one another, influencing their performance (e.g., happier team members tend to be more resilient and perform better; Jiang et al., 2013; Lavy et al., 2015; Meneghel et al., 2016). However, whether mixed (vs. same) emotions expressed in a dyad can influence creative performance remains both a theoretically and practically important question that has yet to be answered.

Building on information processing research and the EASI model, we propose that contrasting emotions expressed by interacting partners (e.g., happy actor and angry partner) has the potential to enhance collective creativity collectively. Classic theories of epistemic motivation suggest that people have a fundamental desire to understand and to be understood (e.g., Swann et al., 1992; Kruglanski et al., 2009). Individuals generally seek a shared understanding with others about their social environment (Echterhoff et al., 2009, 2013). This can occur through people sharing the same expressed emotions in an interaction (e.g., Hatfield et al., 1994; Echterhoff et al., 2013). On the contrary, when people express contrasting emotions in an interaction, the sense of sharedness can be disrupted. This disruption can encourage people to adjust their knowledge and stimulate information processing, increasing mutual efforts for the interacting partners to seek and integrate information from each other (Louis and Sutton, 1991; Echterhoff et al., 2013).

As discussed in the EASI model (Van Kleef, 2010), the emotional expressions of an interacting partner can provide information to the self. When there is a discrepancy in the emotion expressed by the self and the partner, it signals that the two parties might have divergent understandings of in the situation, such as different preferences and expectations. The discrepancy in interpersonal understanding can motivate both parties to seek further information from each other (Louis and Sutton, 1991). Through more extensive information search, it can enhance perspective taking, helping individuals to compare and consider their own views as well as that of the others (Epley et al., 2004; Leung et al., 2008). It can also open new channels for communication, enabling the parties to share information that they would not have shared otherwise (Hoever et al., 2012; Trötschel et al., 2013). By bringing diverse information to the surface that might not be available to the individuals alone, the parties involved are more likely to think outside of the box and integrate seemingly unrelated or even incompatible ideas between the interacting partners. Therefore, they should be more able to collectively generate more ideas and have a better chance to discover creative integrations (Tadmor et al., 2012), resulting in a better collective creative performance.

Taken together, as expressed emotions can shape information search and integration processes between interacting partners, we argue that interpersonal mixed emotions can lead to better collective creative performance. Since this is a new theoretical perspective, we conducted an empirical test in the context of two-party negotiations. This test represents a first step toward establishing the relationship between interpersonal mixed emotion and collective creative performance.

### Understanding the Negotiation Context

Our study investigates whether interpersonal mixed emotions (vs. same emotion) predict a better joint creative performance in negotiation. We chose the context of two-party integrative negotiation for two reasons. First, negotiation is an ecologically valid context as it is common for negotiators to express emotions which can influence negotiation outcomes (Kopelman et al., 2006). Second, the level of joint gains (i.e., the sum of the individual gains in a negotiation) can systematically reflect the extent to which a dyad achieves a creative solution (see Thompson, 1990; Brett, 2007; Maddux et al., 2010; Gunia et al., 2011) and serve as an objective measure of the joint creative performance.

#### Creativity in Negotiation

The link between creativity and negotiation is widely discussed in the literature. Classic negotiation research posits that a significant obstacle for effective negotiation is the lack of insights in the relations between negotiable issues (Raiffa, 1982; Fisher et al., 1991). By default, negotiators tend to come up with a zero-sum solution. They are likely to perceive available resources on the table as fixed and assume a win-lose situation: the gain of one party equates to the loss of the other (Thompson and Hastie, 1990). Having creative insights about the relations between the negotiable issues, however, shift the negotiation away from a win-lose to win-win situation. As such, negotiators are able to integrate multiple issues to generate value and expand the resources on the table (beyond the threshold of a zero-sum solution). The additional value generated is known as “integrative potential.”

Attaining integrative potential involves interpersonal creativity process (Kurtzberg, 1998; Ott et al., 2016). Negotiators need to understand the preference of their partner, bridge seemingly conflicting interests, and break through the default zero-sum mindset to generate creative trade-offs, which often involve conceding on the less important issue(s) to gain value on more important issues. Generation of creative trade-offs yields extra value for both negotiators to claim (Pruitt, 1981; Raiffa, 1982). Mary Parker Follet, an organizational behavior pioneer, illustrated the idea with a story of two sisters and an orange (Fisher et al., 1991). Two sisters fight over an orange and decide to split it in half. One eats the fruit and throw away the peel, whereas the other only uses the peel to bake and throws away the rest of the orange. Each got half of what they wanted. Only if they were able to move away from their default zero-sum mindset to be more creative, connecting their different uses of the orange and realizing that the orange can be split differently (one gets the fruit inside, the other gets the peel; i.e., non-zero-sum), they both would have got 100% of what they wanted. Therefore, being able to detect integrative potential and “creates values” on the table beyond the default zero-sum solution (50–50) is a signature of collective creative performance in negotiation (Kurtzberg, 1998).

The achievement of integrative potential in creating value is reflected in the level of joint gain in the negotiation (Fisher et al., 1991; Hyder et al., 2000). As such, the more creative a dyad is, the more likely it will attain a higher level of joint gains. Moreover, as increased joint gain is due to the added value created by tapping into the integrative potential, the joint gain of the dyad should also exceed the (non-creative) zero-sum threshold. Consistent with this idea, some work has shown that negotiators with higher creativity can achieve higher levels of joint gain, suggesting that joint gain is an important indicator of collective creative performance (Kurtzberg, 1998; Ott et al., 2016).

#### Emotions in Negotiation

Negotiation research has traditionally adopted a behavioral decision perspective (Bazerman, 1998). Relatively little consideration was given to social and emotional factors (see Bazerman et al., 2000). But recently, research has started to unveil the role of emotions in negotiations (Van Kleef et al., 2004b; Lelieveld et al., 2011).

Negotiators’ expressed emotions can affect joint gain. Extant work mainly focused on the displays of anger and happiness (Olekalns and Druckman, 2014). The use of these emotions in negotiations has both advantages and disadvantages (see Olekalns and Druckman, 2014; Hunsaker, 2017). For instance, an angry negotiator tends to appear tough (Van Kleef and De Dreu, 2010). Although the display of anger sometimes elicits more concession from the negotiation counterpart (Van Kleef et al., 2004a,b; Sinaceur and Tiedens, 2006; Van Kleef and De Dreu, 2010; Sinaceur et al., 2011), it can also trigger backlash, resulting in more competition, worse negotiation gains, and poorer future business relationships (Van Kleef and Côté, 2007; Wang et al., 2012; Campagna et al., 2016). On the contrary, a happy negotiator seems easygoing (Wall, 1991). The display of happiness can sometimes lead the counterpart to cooperate, to implement the final agreement, and to be willing to negotiate again in the future (Kopelman et al., 2006; Carnevale, 2008; Mislin et al., 2011; Campagna et al., 2016). However, happiness also signals satisfaction, which can instill a sense of complacency (Wall, 1991). It results in premature closure, which means that the negotiating parties fail to search for all possible ways to maximize integrative potential before closing a deal (Jemison et al., 1986). Indeed, happiness was found to be less effective than anger in eliciting concession makings from the counterpart and sometimes predict lower negotiation gains (Van Kleef et al., 2004a; Sinaceur and Tiedens, 2006; Overbeck et al., 2010).

Other research has also begun to examine contextual variables that influence the effects of anger and happiness on negotiation outcomes (Morris and Keltner, 2000). The contextual factors include time pressure of the negotiation (e.g., Van Kleef et al., 2004b), cultural expectations of the display of emotions (e.g., Adam et al., 2010; Adam and Shirako, 2013), targets of the emotions (e.g., Lelieveld et al., 2011), the level of justification of the use of emotions (e.g., power; Van Kleef et al., 2006), and the changes and complexity of the emotions expressed from a negotiator (Filipowicz et al., 2011; Rothman, 2011). Nevertheless, most of the studies have focused on the impact of only one side of the emotional exchange in an interaction (e.g., the emotion of one party in a two-party negotiation). Despite the surge of valuable research on emotions in negotiation, the impact of mixed emotional expressions of negotiators remains understudied, and its influences on joint outcomes are not well understood (Van Kleef, 2009).

Taking an interpersonal perspective of emotions and drawing from the EASI model (Van Kleef, 2009), we argue that the experience of interpersonal mixed emotion between negotiating parties can have critical interpersonal implications on collective creative performance. As discussed before, interpersonal mixed emotions have the potential to promote more creative performance. The display of conflicting emotions in an interaction (e.g., angry and happiness) can disrupt a common sense of sharedness and signals that the parties involved have divergent understandings of in the situation, enhancing the search and integration of information between the interacting partners (e.g., more perspective taking and more open information-sharing; Louis and Sutton, 1991; Hoever et al., 2012). These processes should enhance creativity, leading the negotiating parties to ponder more deeply about the counterpart’s preferences and motivate them to entertain possible connections of seemingly unrelated issues at the table (Galinsky et al., 2008; Gunia et al., 2011; Trötschel et al., 2011). Consequently, they are more likely to discover integrative potential and attain a more creative joint performance (Gunia et al., 2011), which is indicated by a higher joint gains and joint gains that exceed the (non-creative) zero-sum threshold.

*Hypothesis 1* (H1): mixed-emotion (vs. same-emotion) dyads will achieve higher joint gains.
*Hypothesis 2* (H2): mixed-emotion dyads will achieve a level of joint gain that is above the (non-creative) zero-sum threshold, but not same-emotion dyads.

#### Study Overview

An experiment is conducted to test these hypotheses. We manipulated negotiators’ expressed emotions in a dyadic negotiation that has hidden integrative potential. Given the emphases on anger and happiness in the negotiation literature (Olekalns and Druckman, 2014), we manipulated negotiators’ expression of anger and happiness. Each negotiation dyad had either mixed or same emotions between two interacting partners. We measured their negotiated agreement and calculated joint gains as their level of joint creative outcome. If interpersonal mixed emotions increase creativity in the negotiation, the negotiation dyads with mixed emotions will uncover the integrative potential and reach a more creative solution. Accordingly, we expect that negotiation dyads with mixed emotions will achieve a higher joint gain compared to those with the same emotions. In addition, their level of joint gain should exceed the zero-sum threshold (i.e., the level of joint gain when negotiators split the presumably fixed resources in half; a non-creative solution).

## Materials and Methods

### Participants, Design, and Power

Two hundred and ten undergraduate business majors from a university in Hong Kong participated in the study for course credit or cash payment (112 females, 180 local students, median age = 20, mean_age_ = 19.49, SD_age_ = 0.92). The study involved a face-to-face dyadic negotiation. Before the negotiation, participants were randomly assigned to receive one of two versions of a manipulation that guided them to express either anger or happiness during the negotiation. We assigned the participants into dyads to negotiate with a partner. The assignment was random, except that we tried to form same-gender dyads whenever possible because of known gender differences in negotiation behaviors (Kray et al., 2002; Gunia et al., 2011; Mazei et al., 2015). This resulted in 97 same-gender dyads (92%; *n*_female dyads_ = 52; *n*_male dyads_ = 53).^2^ Critically, the dyad assignment created three emotion compositions between negotiators in a dyad. They were happy-happy (37 dyads), angry-angry (35 dyads), and happy-angry (33 dyads). This design allowed us to compare the outcomes across mixed-emotions (happy-angry) vs. same-emotion (happy-happy, angry-angry) negotiations. A sensitivity power analysis showed the number of dyads (*n* = 105) gave us 80% power to detect a minimum medium effect size (*d* = 0.59; two-tailed with *p*-value less than 0.05) of a difference between mixed- and same-emotion negotiations (Faul et al., 2009).

### Emotion Manipulation (Independent Variable)

Before the negotiation took place, participants received an information package to prepare for the negotiation individually. An emotion manipulation was embedded in the package. As part of the preparation, the manipulation was presented as a guide to help participants negotiate effectively. Happiness and anger were selected as the target of the manipulation because they are important emotions in negotiation (Sinaceur and Tiedens, 2006; Adam and Shirako, 2013). The manipulation summarized recent findings showing the utility of either happiness or anger in negotiation. It encouraged participants to express the emotion to increase negotiation effectiveness, and provided several sample phrases taken from past research to communicate the emotion (Van Kleef and Côté, 2007; Sinaceur et al., 2011; Adam and Brett, 2015). See Appendix for verbatim manipulation materials.

In the *angry* condition, participants read:

“…angry statements in negotiation induces concession-making and cooperation, such as ‘this offer makes me really angry; I think I will offer…,’ ‘this offer is really getting on my nerves! I’m not happy at all. It makes me irritated,’ ‘This is not serious! I’m fed up with this. This negotiation pisses me off” and ‘I’m very angry now.’

In the following negotiation, you can try to use your emotions to obtain concessions and negotiate. You can use angry statements to express your feelings…”

In the *happy* condition, participants read:

“…happy statements in negotiation induces concession-making and cooperation, such as ‘I am happy with this offer; I think I will offer…,’ ‘this offer pleases me much! I’m very happy. It makes me feel good,’ ‘This is really cool! I’m delighted about this. I’m very happy with this negotiation,’ and ‘I’m very happy now.’

In the following negotiation, you can try to use your emotions to obtain concessions and negotiate. You can use happy statements to express your feelings…”

After reading the manipulation, participants were provided with some space on a piece of paper to practice. They thought about how they might express the emotion and were encouraged to write the phrases down as their notes.

### Negotiation Simulation and Joint Gains (Dependent Variable)

Participants had 35 min to complete a two-party standardized negotiation simulation called “The Sweet Shop”.^3^ In this negotiation, participants were the owners of an ice-cream and a bakery shop who were trying to form a plan to expand and share space at a new store location. The negotiation involved a total of six issues. Four were core issues (i.e., temperature, staffing, maintenance, and design) that must be decided to reach an agreement; two were optional issues (i.e., website design and delivery plan). For each issue, there were two to five options with varying amounts of points. Higher points indicated a better option, and a negotiator’s gain was calculated by the sum of points achieved on all issues.

The performance of a negotiation dyad was calculated by the sum of the two negotiators’ individual gains, called joint gains. The (non-creative) zero-sum threshold in the negotiation simulation was 16,100. There was hidden integrative potential for negotiators to create more values if they were able to break away from the default zero-sum mindset. Two of the core issues, staffing and design, were integrative issues, which had the potential to create a higher joint value. If negotiators could connect these two seemingly unrelated issues and realize the integrative potential, they could generate extra value by trading off the issue of lower priority for themselves in exchange for a better option in an issue of higher priority (Pruitt, 1981; Raiffa, 1982; Fisher et al., 1991). The more negotiators unlocked this hidden value at the negotiation table, the more they could increase their joint gains (up to a maximum of 19,700). Joint gains served as our measure of collective creative performance (it ranged from 8,000 to 19,700). More creative dyads should be more likely to achieve a joint gain that was higher than the zero-sum threshold (i.e., 16,100).^4^

### Manipulation Check

To check whether the emotion manipulation was effective, after the negotiation (before their partner’s individual gain was revealed) participants were asked, “during the negotiation, to what extent did you and your partner express the following emotions?” On a scale from 1 (*Not at all*) to 7 (*Very much*), they indicated the extent to which they *themselves* expressed “happy” and “angry” during the negotiation. They also used the same scale to indicate the extent to which their *partner* has expressed “happy” and “angry.” These ratings were used to examine whether or not the manipulation had affected the emotions participants expressed from their own perspective and from their partner’s observation.

### Exploratory Variables (Covariates)

In addition, we sought to explore whether contrasting emotions expressed *between* negotiators (interpersonal mixed emotions) have unique predictive validity over and beyond contrasting emotions expressed *within* negotiators (the conventional conceptualization of mixed emotions; Larsen et al., 2001; Fong, 2006; Rothman and Northcraft, 2015). To do so, we utilized participants’ self-reported ratings of their levels of expressed anger and happiness in the negotiation—the manipulation check responses. For each participant, we used the attitude ambivalence formula to calculate a mixed-emotions score: (Rating_anger_ + Rating_happiness_)/2 -| Rating_anger_ - Rating_happiness_ | (Thompson et al., 1995). The first part of the formula captures average intensity and the second part captures the level of similarity (between the ratings of anger and happiness). In essence, a higher score signifies a stronger experience of both anger and happiness simultaneously. We then averaged the scores within a dyad to form the dyad’s within-person mixed-emotions score, which served as a control variable in exploratory analyses.

At the end of the study, we also measured demographic and ancillary information, such as gender and how well participants knew their partner before the interaction, which used the question of “How well do you know your negotiation partner inside and outside of class?” on a scale from 1 (*Not at all*) to 5 (*Extremely well*). More than half of the participants (112 out of 210) reported not knowing their partner before the negotiation (*M* = 1.87, SD = 1.12). As gender and prior relationship with the partner may influence the outcomes in the negotiation exercise (e.g., Mazei et al., 2015), we coded gender of the dyad (1 = female, 0 = male) and familiarity with the negotiation partner as potential control variables for exploratory analyses.

## Results

To start with, we analyzed the manipulation check responses using repeated-measures ANOVAs. There was a significant interaction between Manipulation (Angry vs. Happy; between-subjects) × Expressed Emotions (Angry vs. Happy; within-subjects) on participant’s *own* ratings of expressed emotions, *F*(208) = 4.55, *p* = 0.034, $\eta_{p}^{2}$ = 0.02. This suggests that the manipulation affected the self-reported expressed emotions of the participants during the negotiation. Specifically, those in the angry condition found themselves expressing more anger (*M* = 2.17, SD = 1.41) than those in the happy condition (*M* = 1.72, SD = 1.02), *t*(208) = 2.59, *p* = 0.010, $\eta_{p}^{2}$ = 0.03. There was no significant difference in the level of expressed happiness between the angry (*M* = 4.95, SD = 0.88) and happy conditions (*M* = 5.07, SD = 0.92) but they were in the expected direction. The same analysis was conducted using the ratings from their partner’s observation. We found no significant Manipulation × Expressed Emotions interaction, *F*(208) = 2.37, *p* = 0.126, $\eta_{p}^{2}$ = 0.01. Nonetheless, the pattern of partners’ ratings of the participant’s anger (*M*_angry condition_ = 2.03, SD = 1.29, *M*_happy condition_ = 1.78, SD = 1.22) and happiness (*M*_angry condition_ = 4.68, SD = 1.30, *M*_happy condition_ = 4.84, SD = 0.99) appeared in the expected directions and are comparable to that of participants’ own ratings, an observation we will revisit in the discussion. In short, there was some evidence demonstrating that the manipulation affected emotions expressed in the negotiation. Subsequently, we tested our two hypotheses.

**FIGURE 1:**
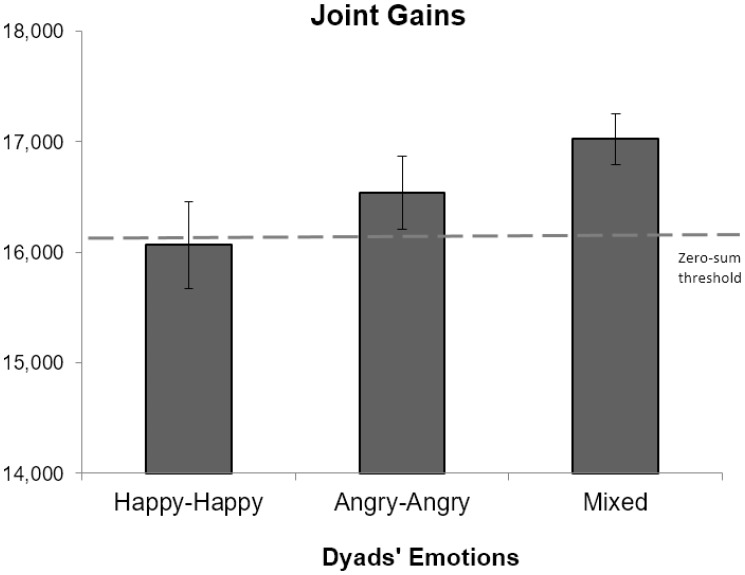
Joint gains as a function of the dyad’s emotion composition. Error bars are means ±1 SE.

Hypothesis 1 predicted that the mixed-emotions dyads (happy-angry) would have higher joint gains (i.e., more creative integration outcomes) compared with the same-emotion dyads (happy-happy or angry-angry). To test the hypothesis, we conducted an ANOVA using dyadic emotion compositions (three-levels: happy-happy, angry-angry, happy-angry) as the predictor and joint gains (the sum of the individual gains in a dyad) as the outcome.^5^ Mean differences across conditions are presented in Table 1. As illustrated in Figure 1, joint gains significantly differed as a function of the dyad’s emotion compositions, *F*(103) = 4.18, *p* = 0.043, $\eta_{p}^{2}$ = 0.04. Planned contrast analysis revealed that the mixed-emotions dyads showed a trend of yielding higher joint gains (*M* = 1,7024.24, SD = 1,303.85) compared with the same-emotion dyads (happy-happy and angry-angry; *M* = 16,297.22, SD = 2,198.33), *F*(103) = 3.10, *p* = 0.081, $\eta_{p}^{2}$ = 0.03, *d* = 0.37, although the effect did not reach the conventional level of statistical significance.^6^ Results are presented in Table 2.

**Table 1:** Means, SDs of study variables.

	Mean (SD)			*F*	*df*	*p*	η^2^
		
	Happy-Happy	Angry-Angry	Mixed-Emotions				
1. Joint gain	16,067.57 (2,392.47)^b^	16,540.00 (1,978.15)^a,b^	17,024.24 (1,303.85)^a^	4.18	103	0.043	0.04
2. Individual gain^†^	8,068.92 (1,690.76)^a^	8,270.00 (1,569.53)^a^	8,512.24 (1,554.56)^a^	2.85	208	0.093	0.01
3. Dyadic within-person mixed-emotions score	-0.26 (1.18)^b^	0.36 (1.20)^a^	0.43 (1.44)^a^	5.27	103	0.024	0.05
4. Within-person mixed-emotions score^†^	-0.26 (1.51)^b^	0.36 (1.84)^a^	0.43 (1.74)^a^	5.91	208	0.016	0.03


**N* = 211 (105 dyads). Dyads: Happy-Happy (*n* = 37), Angry-Angry (*n* = 35), and Mixed-Emotions (Happy-Angry, *n* = 33). ^†^Individual-level variables. Means that do not share the same superscript differ from each other at the level of *p* < 0.05.*

**Table 2:** Test of Hypothesis 1 and exploratory analyses: *T*-test and ANCOVAs.

Model		*B*	SE	*t*	*df*	*p*	95% CI	$\eta_{p}^{2}$
**Test of Hypothesis 1**					
1.	Mixed-emotions	727.03^†^	412.98	1.76	103	0.081	[-92.03, 1,546.08]	0.03
**Exploratory ANCOVA**					
2.	Intercept	16,406.32^∗∗∗^	336.30	48.78	94	<0.001	[15,738.59, 17,074.06]	0.96
	Mixed-emotions	659.73	425.76	1.55	94	0.125	[-185.61, 1,505.08]	0.02
	Gender	-86.24	404.48	-0.21	94	0.832	[-889.35, 716.87]	0.00
3.	Intercept	16,026.32^∗∗∗^	448.30	35.75	102	<0.001	[15,137.12, 16,915.52]	0.93
	Mixed-emotions	682.84	418.69	1.63	102	0.106	[-147.62, 1,513.31]	0.03
	Familiarity with partner	151.79	214.91	0.71	102	0.482	[-274.48, 578.05]	0.00
4.	Intercept	16,308.35^∗∗∗^	229.12	71.18	102	<0.001	[15,853.89, 16,762.82]	0.98
	Mixed-emotions	831.24^∗^	412.63	2.01	102	0.047	[12.788, 1,649.69]	0.04
	Dyadic within-person mixed-emotions	-267.12^†^	148.28	-1.80	102	0.075	[-561.24, 26.99]	0.03


*Dyad *N* = 105. Mixed-emotions (1 = mixed-emotions, 0 = same-emotions), gender (1 = female, 0 = male). ^∗∗∗^*p* ≤ 0.001, ^∗^*p* ≤ 0.05, ^†^*p* ≤ 0.08.*

We then conducted exploratory analyses to test the robustness of the hypothesized effect when entering other variables as controls. Results are presented in Table 2. Controlling for gender and familiarity of partner did not change the general pattern of results. However, controlling for within-person mixed emotions reduced the significance level of the contrast between mixed-emotions (vs. same-emotion) on joint gains, *p* = 0.047, $\eta_{p}^{2}$ = 0.04, *d* = 0.42 (Estimated mean_mixed-emotions_ = 17,095.71, SD = 1,303.85, Estimated mean_same-emotion_ = 16,264.47, SD = 2,198.33). The finding seems to suggest that intrapersonal and interpersonal mixed emotions might have independent effects on joint creative performance. The main and exploratory analyses together provided partial support to Hypothesis 1.

Hypothesis 2 predicted that mixed-emotions dyads’ joint gains should be higher than the (non-creative) zero-sum threshold (i.e., 16,100). This rapothesis can be tested with two approaches. The more straightforward approach is to use a one-sample *t*-test, which tests the difference between the mean level of joint gains of the mixed-emotions dyads against the zero-sum threshold. The result showed that mixed-emotions dyads’ levels of joint gains indeed significantly exceeded the zero-sum threshold (924.24 points higher than the threshold), *t*(32) = 4.07, *p* < 0.001, 95% CI[461.92, 1,386.57]. Neither angry dyads’ joint gains, *t*(34) = 1.32, *p* = 0.197, nor happy dyads’ joint gains exceeded this level, *t*(36) = -0.08, *p* = 0.935. Results provided initial support to Hypothesis 2.

An alternative approach to testing Hypothesis 2 is to use an intercept test in multiple regression. This method has two main advantages. First, it retains the entire sample in the test, even though the condition of interest is only the mixed-emotions condition. Second, it allows (control) variables in the model for exploratory analyses. To do so, we first created two dummy-coded variables for the same-emotions conditions: Happy-happy (1 = happy-happy; 0 = rest) and angry-angry (1 = angry-angry; 0 = rest). We then created a new joint gain variable by subtracting the points of the zero-sum threshold (16,100) from dyads’ level of joint gain. This variable represented the degree to which a dyad’s joint gain differed from the zero-sum threshold (i.e., 0 = the same; a positive/negative value means higher/lower than the threshold). By regressing the new joint gain variable on the two same-emotions condition dummy variables, the intercept test in the statistical model was the significance test of whether the level of joint gains in mixed-emotions dyads differed from the zero-sum threshold (zero). Results are presented in Table 3. The intercept test was significant, *p* = 0.016. Including different control variables in the model did not alter the pattern of this result. Taken together, both the one-sample *t*-test and intercept test in multiple regression suggested that mixed-emotions dyads achieved a level of joint gains that was significantly above the zero-sum threshold. The findings supported Hypothesis 2, suggesting that interpersonal mixed emotion results in exceedingly high level of joint gain, an indicator of creative performance.

**Table 3:** Test of Hypothesis 2 and exploratory analyses: multiple regression models of the amount of joint gains minus the zero-sum threshold (16,100).

Model		*B*	SE	*t*	*df*	*p*	95% CI	$\eta_{p}^{2}$
**Test of Hypothesis 2**		
1.	Mixed-emotions (Intercept)	924.24^∗^	341.92	2.46	102	0.016	[186.36, 1,760.78]	0.06
	Happy-happy	-956.67^∗^	470.29	-2.03	102	0.045	[-1,889.49, -23.86]	0.04
	Angry-angry	-484.24	476.58	-1.02	102	0.312	[-1,429.54, 461.06]	0.01
**Exploratory analyses**		
2.	Mixed-emotions (Intercept)	973.58^∗^	396.41	2.46	93	0.016	[186.36, 1,760.78]	0.06
	Happy-happy	-904.05^†^	480.82	-1.88	93	0.063	[-1,858.87, 50.77]	0.04
	Angry-angry	-362.20	505.45	-0.72	93	0.475	[-1,365.92, 641.52]	0.01
	Gender	-101.77	404.33	-0.25	93	0.802	[-904.69, 701.15]	0.00
3.	Mixed-emotions (Intercept)	1,057.98^∗∗^	342.84	3.09	101	0.003	[377.87, 1,738.09]	0.09
	Happy-happy	-1,169.93^∗^	474.47	-2.47	101	0.015	[-2,111.16, -228.71]	0.06
	Angry-angry	-507.37	469.34	-1.08	101	0.282	[-1,438.42, 423.68]	0.01
	Familiarity with partner	-309.71^∗^	150.54	-2.06	101	0.042	[-608.34, -11.08]	0.04
4.	Mixed-emotions (Intercept)	1,057.98^∗∗^	342.84	3.09	101	0.003	[377.87, 1,738.09]	0.09
	Happy-happy	-1,169.93^∗^	474.47	-2.47	101	0.015	[-2,111.16, -228.701]	0.06
	Angry-angry	-507.37	469.34	-1.08	101	0.282	[-1,438.42, 423.68]	0.01
	Dyadic within-person mixed-emotions	-309.71^∗^	150.54	-2.06	101	0.042	[-608.34, -11.08]	0.04


*Dyad *N* = 105. The intercept test: the significance test of whether the level of joint gains of mixed-emotions dyads was significantly different from the zero-sum threshold. ^∗∗^*p* ≤ 0.01, ^∗^*p* ≤ 0.05, ^†^*p* ≤ 0.08.*

Lastly, as supplemental analyses, we tested whether the control variables would moderate the effect of dyadic emotions (mixed vs. same) on joint gains. Multiple regression analyses showed that none of the interaction terms were individually significant: gender of the dyad, *p* = 0.639; familiarity with partner, *p* = 0.750, and dyadic within-person mixed emotions, *p* = 0.305. These results suggest that the effect of interpersonal mixed (vs. same) emotions on collective creativity may generalize across gender composition, familiarity with the partner, and the experience of intrapersonal mixed emotion.

## Discussion

We theorized that interpersonal mixed emotions might facilitate collective creative performance and tested our hypotheses in a negotiation context. There was partial evidence suggesting that negotiators who were in a mixed-emotion dyad (i.e., angry-happy) performed better than those in a same-emotion dyad (i.e., happy-happy, angry-angry; Hypothesis 1). Additionally, mixed-emotion dyads, on average, achieved a high level of joint gain that exceeded the (non-creative) zero-sum threshold whereas same-emotion dyads did not (Hypothesis 2). Exploratory analyses controlling for dyads’ within-person mixed emotions did not alter the pattern of results, suggesting that increased collective creative performance was likely due to mixed emotions expressed between (not within) negotiators. Together, these findings shed light on the possibility that interpersonal mixed emotions increase joint creative performance and make theoretical and practical contributions to at least three bodies of literature.

First, the results enrich the creativity literature by uncovering the interpersonal impact of mixed emotions on creativity. The study of creative thinking has conventionally focused on intrapersonal processes (Barron and Harrington, 1981; Gong et al., 2009, 2017; Anderson et al., 2014). Little research has examined how to foster creativity between two (or more people) and what factors increase collective creative performance. The current research adds knowledge to fill this gap and illuminates the hidden role of contrasting (vs. converging) emotions in dyadic creativity process. It also offers empirical evidence that suggests that interpersonal mixed emotions can be one potential avenue for improving a collective creative outcome.

Second, the current work extends the emotion literature. By integrating the classic feeling-as-information theory (Schwarz and Clore, 1996; Forgas, 2000) and the emotion-as-social information model (Van Kleef, 2009), we argued that the interplay between self- and partner-expressed emotions in interpersonal situations could have social consequences. This theoretical integration unlocks a novel perspective for emotion research to examine whether contrasting and converging interpersonal emotions matter. Additionally, this study provides an empirical example of this perspective. We hypothesized and found some initial support that contrasting (vs. converging) interpersonal emotions have implications for dyadic creativity. This generates new insights into understanding the diverse impact of emotions in interpersonal contexts. Together with the existing literature, this work encourages emotion research to examine rich processes and outcomes of interpersonal emotions that have yet to be uncovered.

Third, this research adds to the organizational psychology literature. Social interactions are an integral part of an organization; organizational members need to manage their own emotions and sometimes the emotions of others (Sy et al., 2005; Barger and Grandey, 2006). However, interpersonal processes of emotions at work are still not well understood. Our findings advance the literature by showing that the understanding of interpersonal mixed emotions is critical and has practical implications. In particular, the study showed that interpersonal mixed emotions matter for collective creativity. Creative work often involves social interactions (e.g., negotiating an agreement, teamwork; Kurtzberg and Amabile, 2001). Facilitating creativity in these collective situations is critical (Liu et al., 2017). Our study suggests that one important factor is the interpersonal experience of mixed emotions. Consistent with past research on within-person mixed emotions (Fong, 2006), we found that mixed emotions between two people may also help workers realize potential interconnections between unrelated ideas and result in a more creative outcome. This finding opens a new door for organizations to understand and potentially utilize emotions as a strategy to facilitate conflict resolution and increase organizational effectiveness. For example, organizations traditionally consider emotions as impediments to rationality in decision-making (see Bazerman and Neale, 1994). Yet our work highlights that emotions should not be suppressed because they may lead to beneficial interpersonal outcomes. Practically, organizations may benefit from strategic use of emotions, developing emotion regulation training or priming methods and encouraging the expressions of emotions at work in proper contexts (e.g., ones that require creativity).

### Limitations and Future Directions

We draw attention to several limitations of the study and recommendations for future research. To begin, as we tried to manipulate emotional expressions across dyads systematically, this study implicitly assumes that participants’ expressed emotions are constant throughout the interaction. However, this might not be true in reality. Emotions can change over the course of a social interaction and the emotions expressed by interacting partners are mutually influential. A person’s emotion can influence and be influenced by the partner (Van Kleef, 2009). Future studies will benefit from more sophisticated methods in capturing the temporal dynamics of emotion during social interactions. For instance, an audio or video recording of the negotiation allows researchers to code emotion-related verbal and non-verbal behaviors (Adair and Brett, 2005; Martinovsky, 2015). These temporal techniques will generate more insights into not just the content of the emotional exchange, but also a dynamic view of the emotional processes throughout the negotiation.

A more nuanced approach is also needed to understand the complexity in the dimensions of contrasting emotions fully. Both the dimensions of felt and expressed emotions are essential. They are often interdependent. Felt emotions can affect expressed emotions, and *vice versa*. They often move in the same direction (e.g., Van Kleef et al., 2004a). However, it is also possible that the two dimensions of emotions operate independently, which means that felt emotion may not be reflected in expressed emotion (Grandey, 2003). Based on our theorizing, the contrast in any combinations of felt and expressed emotions between partners would suffice to enhance creative performance as long as they create conflicting information. However, our study did not measure felt emotions during the negotiation. This assumption remains an empirical question for future research to explore.

In terms of methods, the results seem to suggest that our manipulation of emotional expressions was successful, but the effect was weak, especially from the standpoints of the observers (i.e., the partners). Although there was evidence from the participants’ own perspective that the (anger/happiness) manipulation changed their (angry/happy) emotional expression, the partners (observers) only showed trends in detecting the emotion expressed by the focal participants. The magnitude of the effects detected by the partners was much weaker. This relative difference between the participants and their partners might be natural as the participants were not explicitly told to observe and assess their partner’s emotion during the negotiation. It was also possible that observers might be less precise in inferring partner’s emotion as their own emotional experiences can also influence their perceptions. This discrepancy in expressing and observing emotions and its influence on creativity merit further research. In addition, future research should also consider ways to manipulate emotional experiences, for instance, employing multiple means to activate emotions (e.g., recall task and a computer-mediated negotiation with default messages; Van Kleef et al., 2004a; Fong, 2006) and encouraging multiple dimensions of emotion expressions (e.g., tone of voice, body language; Martinovsky, 2015). Additionally, while the context of negotiation has offered both ecological and construct validity for the study of collective creativity (Thompson, 1990), future studies should consider the use of other creativity tasks and interpersonal contexts to replicate the results (e.g., unusual use task, creative problem-solving in teams; Tadmor et al., 2012; Tsai et al., 2012).

As the current work provides evidence for the general effect of interpersonal mixed emotions on creativity, it is also essential for future work to examine the mechanisms of the effects. One major limitation of the study is the lack of process measurement that captured dynamic characteristics of the interaction. There is much we do not know about how interpersonal creativity occurs. For instance, though we have speculated that interpersonal mixed emotions affect creativity through enhancing information search and integration processes (e.g., perspective taking), there is no direct evidence to demonstrate how the effect exactly occurs. There is also much to learn about the dynamics between emotion expressions and reactions. For example, how exactly negotiators utilized a specific emotion to trigger specific reactions and how they feel and react in response (Van Kleef et al., 2010). Further, emotions can evolve as the negotiation continues. It is important to understand the impact of momentary emotions to answer deeper questions such as how frequently people alternate emotional strategies (Olekalns and Druckman, 2014), how the order of occurrence of emotions may affect negotiation outcome (e.g., angry first, happy after; Sinaceur et al., 2013), and how the synchrony and asynchrony of emotions expressions affect interpersonal creativity. These nuances will bring new insights into the understanding of emotional dynamics and interpersonal outcomes. These questions about mechanisms, as discussed above, will require future research to use more sophisticated study design, such as utilizing video-recording and physiological measures (Adair and Brett, 2005; Ben-Shakhar et al., 2007), to answer.

In addition, future research should also test boundary conditions to understand when the effect might be strengthened or reduced. First, individual differences may moderate the effect of interpersonal mixed emotions. For example, people can differ in their level of openness to experience (Kaufman, 2013). It is possible that open-minded people might be more willing to engage in the search of new and different information from their interacting partner, perhaps like situations that elicit interpersonal mixed emotions, and attain the creative benefits. Yet, it is also possible that due to desensitization to extraordinary experiences over time, open-minded people might be less stimulated by repeated interpersonal mixed emotions and no longer find it thought-provoking.

Second, the way emotions are expressed may play a role. One dimension of emotions is the degree to which they are authentic. It is possible that authentic emotional expressions are more impactful in influencing partners’ reactions and disingenine emotional expressisons are seen as instrumental and are discounted by the partner, potentially causing negative downstream consequences (e.g., reducing trust; see Campagna et al., 2016). Because our study directly manipuated emotional expressions, we do not know to what extent participants felt the emotions manipuated and how exactly the partner responded, which again requires measures sensitive to the processes to explore. Another dimension is the level of intensity of emotional expressions (Russell, 1980). The same content of emotions can be expressed in different levels of intensity (e.g., a happy smile vs. a ecstatic scream of joy). Theoretically, more intense emotions should have a stronger influence on the partner’s response. This could mean that increasing the intensity of interpersonal mixed emotions could likely increase the level of interpersonal creativity, and yet given the lack of research on mixed emotions in interpersonal interactions, this moderating effect needs future research to examine.

Third, contextual factors can alter the relations between interpersonal emotions and outcomes. For instance, some situations motivate people to process information more intensely than others. Contextual variables such as low time pressure, low cognitive load/complexity of the task, and low power tend to increase information-processing motivation and should amplify the effect of interpersonal mixed emotions (Van Kleef, 2009). Additionally, contexts may also matter for the functions of emotions (Morris and Keltner, 2000). For instance, positive emotions like displays of happiness can facilitate more constructive interactions in negotiation (e.g., increases cooperation, promotes future business relationship, enhance willingness to implement the final deal; Kopelman et al., 2006; Mislin et al., 2011). However, happiness can also lead to complacency in negotiation, leading to fewer concessions from the partner and more premature closure (e.g., Sinaceur and Tiedens, 2006; Overbeck et al., 2010). This raises interesting questions about how the interpersonal context may moderate the implications of emotions. In our study, happy-happy dyads achieved the lowest joint gains, which seems to suggest that interpersonal displays of happiness could led to more complacency and was not as effective as mixed emotions in inspiring the search for integrative potential. Nonetheless, it is possible that in other contexts, such as a more competitive distributive negotiation, displays of happiness may be useful in inducing more cooperative exchanges and rapport with the partner (Kopelman et al., 2006). These are exciting nuances that future research will benefit from manipulating the negotiation contexts and measuring subjective outcomes (e.g., trust, relationship satisfaction; Kung et al., 2018) to more fully understand the moderating role of contexts.

Finally, our study is also limited by its sample size and diversity. A larger sample is needed to detect the effect of an interpersonal mixed- (vs. same-) emotions effect more reliably (based on the observed effect size, *d* = 0.37, this would be about 232 dyads; Faul et al., 2009). Additionally, our sample comprised of Hong Kong undergraduates. We do not know whether the results will generalize to other populations that differ in age and culture, for instance. This is especially important because cultures differ in display rules and ideal intensity of emotion expression (Matsumoto, 1990, 1993; Tsai et al., 2007). Emotions that are contrasting in one culture might not be as contrasting in another; hence, the same interpersonal mixed emotions may have varying effects across cultures. Culture influences the extent to which people see conflicting information as contradictory (e.g., dialectical thinking; Miyamoto and Ryff, 2011). In cultures that tend to be less tolerant of apparent contradictions (e.g., North American cultures; Peng and Nisbett, 1999), interpersonal mixed emotions may disrupt the sense of sharedness and stimulate information integration processes more powerfully, resulting in a stronger effect on creativity. These cultural differences and nuances are interesting to explore and critical for the generalization of the findings.

## Conclusion

To conclude, this article proposed and tested that interpersonal mixed emotions have implications for collective creativity. They facilitated the generation of creative solutions in negotiation. This finding have both theoretical and practical implications, advancing research on creativity, emotion, and organizational behavior. Creative challenges often involve social interactions. As individuals and organizations better understand the interpersonal dynamics of emotion, we can unlock creative potential for more optimal conflict resolution and organizational effectiveness.

## Ethics Statement

This study was carried out in accordance with the recommendations of the Committee on Research Practices: Human Participants Research Panel at the Hong Kong University of Science and Technology with written informed consent from all subjects. All subjects gave written informed consent in accordance with the Declaration of Helsinki. The protocol was approved by the Committee on Research Practices: Human Participants Research Panel at the Hong Kong University of Science and Technology.

## Author Contributions

Both authors conceptualized the idea and collected the data. FK analyzed the data and drafted the manuscript. MC provided critical feedback on the manuscript.

## Conflict of Interest Statement

The authors declare that the research was conducted in the absence of any commercial or financial relationships that could be construed as a potential conflict of interest.

## Footnotes

1 Self- and partner-expressed emotions can also differ in that they complement each other (e.g., one partner shows anger, the other shows sadness, which are reciprocal positive and negative emotions; Lelieveld et al., 2012). This is conceptually different from contrasting emotions, which is the focus of the current study. Traditionally, mixed emotions refer to conflicting emotions (see Larsen and McGraw, 2014). Complementary emotions do not invoke a conflicting experience, and therefore, are not incorporated in the current theorization.

2 Same-gender and mixed-gender dyads did not differ in their joint gains, *p* = 0.292, and therefore is not discussed further.

3 negotiationandteamresources.com

4 Two dyads did not reach an agreement. They received the standard 8,000 points (same as their reservation price stated on the information package) (Aslani et al., 2016). To maximum statistical power, we included them in the analysis; however, including them or not did not change the pattern of the results.

5 Supplemental individual-level analysis using the Actor-Partner Interdependence Model (Kenny et al., 2006) showed convergent results; the Actor × Partner interaction was marginally significant, *b* = -171.33, SE = 8.49, *t*(102) = -1.74, *p* = 0.085 95% CI[-366.68, 24.21]. In particular, negotiators in a mixed-emotion dyad achieved higher individual gains (*M* = 8,512.12, SD = 1,356.27) compared to those in a same-emotion dyad (*M* = 8,166.67, SD = 1,630.38).

6 Exploratory pairwise comparisons using Fisher’s Least Significant Difference (LSD) revealed a significant difference in joint gains between mixed-emotions vs. happy dyads, *p* = 0.045, 95% CI[23.86, 1,889.49]; other comparisons were not significant, *p* > 0.309.

## APPENDIX

### Emotion Manipulation: Anger Condition

#### Negotiation Strategy

Getting concessions and a successful deal in a negotiation can often be very difficult; prior to the negotiation, knowing what effective negotiation strategy you can employ to achieve those is therefore very important to your negotiation success. Although some conventional business wisdom may suggest that negotiators should be completely rational and should not show any emotions, research in the past have in fact shown that emotional reactions in negotiation are the triggers of effective negotiation. By unpicking the role of emotion, scientific research has demonstrated that one particular emotion plays an extraordinary role in helping negotiators to make a desirable deal, and that is anger.

Anger was found inducing concession-making and cooperation of the other party in negotiations. It is because emotional experience is not only an intrapersonal experience, but also it conveys interpersonal meanings in negotiation. Instead of framing offers only in numerical terms, framing offers in terms of how you feel angry about it conveys additional social signals to the other party that encourages concession and cooperation. For example, a recent series of studies by Sinaceur et al. (2013) published in Journal of AppliedPsychology have shown angry statements in negotiation induces concession-making and cooperation, such as “*this offer makes me really angry; I think I will offer…*”, “*this offer really getting on my nerves! I’m not happy at all. It makes me irritated*,” “*This is not serious! I’m fed up with this. This negotiation pisses me off*” and “*I’m very angry now*.”

In the following negotiation, you can try to use your emotions to obtain concessions and negotiate. You can use angry statements to express your feeling. When expressing your emotions, target your anger toward the negotiation and the offers, but not the other person. As a practice, below is some space for you to think in advance about how you may want to express anger in the upcoming negotiation. You may or may not model after the example sentences above, and during the negotiation, try to make your statements as natural as possible.

### Emotion Manipulation: Happy Condition

#### Negotiation Strategy

Getting concessions and a successful deal in a negotiation can often be very difficult; prior to the negotiation, knowing what effective negotiation strategy you can employ to achieve those is therefore very important to your negotiation success. Although some conventional business wisdom may suggest that negotiators should be completely rational and should not show any emotions, research in the past have in fact shown that emotional reactions in negotiation are the triggers of effective negotiation. By unpicking the role of emotion, scientific research has demonstrated that one particular emotion plays an extraordinary role in helping negotiators to make a desirable deal, and that ishappiness.

Happiness was found inducing concession-making and cooperation of the other party in negotiations. It is because emotional experience is not only an intrapersonal experience, but also it conveys interpersonal meanings in negotiation. Instead of framing offers only in numerical terms, framing offers in terms of how you feel happy about it conveys additional social signals to the other party that encourages concession and cooperation. For example, a series of studies by Sinaceur et al. (2013) published in Journal of Applied Psychology recently have shown happy statements in negotiation induces concession-making and cooperation, such as “*I am happy with this offer; I think I will offer…*,” “*this offer pleases me much! I’m very happy. It makes me feel good*,” “*This is really cool! I’m delighted about this. I’m very happy with this negotiation*” and “*I’m very happy now*.”

In the following negotiation, you can try to use your emotions to obtain concessions and negotiate. You can use happy statements to express your feeling. When expressing your emotions, target your happiness toward the negotiation and the offers, but not the other person. As a practice, below is some space for you to think in advance about how you may want to express happiness in the upcoming negotiation. You may or may not model after the example sentences above, and during the negotiation, try to make your statements as natural aspossible.
